# Supplementary material for: Boardgames as an innovative approach to promote life skills and well-being among inmates: A scoping review protocol
Source: PLoS One. 2024 Feb 29;19(2):e0298728. doi: 10.1371/journal.pone.0298728 (PMC10903794; doi:10.1371/journal.pone.0298728)
Supplement: S1 Checklist — (PDF) [file pone.0298728.s001.pdf]

PRISMA-P (Preferred Reporting Items for Systematic review and Meta-Analysis Protocols) 2015 checklist: recommended items to address in a systematic review protocol*

Section and topic	Item No	Checklist item
**ADMINISTRATIVE INFORMATION**		
Title:		*Boardgames as an innovative approach to promote life skills and well-being among inmates: A scoping review protocol.*
Identification	1a	*Study protocol of a scoping review*
Update	1b	N.A.
Registration	2	N.A.
Authors:		
Contact	3a	*Maria Giuliana Solinas Corresponding author, Department of Biomedical Sciences, University of Sassari, Via Padre Manzella, 4, 07100 Sassari, Italy; gsolinas@uniss.it*<br>*Carlo Andrea Pensavalle (CAP), Department of Chemistry, Physics, Mathematics, and Natural Sciences, University of Sassari, Sardinia, Italy; pensa@uniss.it*<br>*Christian Gardoni, Department of Primary Education, European University of Rome, Via degli Aldobrandeschi, 190, 00163 Rome, Italy; christian.gardoni@unier.it*<br>*Gabriele Giorgi, Department of Human Sciences, European University of Rome, Via degli Aldobrandeschi, 190, 00163 Rome, Italy; gabriele.giorgi@unier.it*<br>*Tiziano Antognozzi, AXES – Laboratory for the Analysis of Complex Economic Systems, IMT School for Advanced Studies Lucca, Piazza S. Ponziano, 6, 55100 Lucca, Italy, tiziano.antognozzi@imtlucca.it*<br>*Federico Alessio , Business@Health Laboratory, European University of Rome, Via degli Aldobrandeschi, 190, 00163 Rome, Italy; federico.alessio@unier.it*
Contributions	3b	*CAP is the guarantor; all authors contributed equally to the design of the review and writing of the protocol*
Amendments	4	N.A.
Support:		
Sources	5a	*The scoping review is funded by "Fondo di Ateneo per la Ricerca, University of Sassari, 2020"*
Sponsor	5b	*University of Sassari, Italy.*
Role of sponsor or funder	5c	*None*
**INTRODUCTION**		
Rationale	6	*Current research suggests that boardgame activities can facilitate learning and engagement, providing a stimulating experience that capitalizes on the pleasure centres of our brain. Existing literature on boardgames in therapeutic contexts is extensive, but more research is needed to evaluate their efficacy as innovative approaches for positive effects in other populations, including inmates. Incarcerated individuals face numerous mental and physical health difficulties, often due to the lack of recreational or cultural activities as well as training opportunities in prison. For young offenders especially, the*

		learning environment must be different from formal schooling, and those with special education needs face additional difficulties in developing life skills.
Objectives	7	*The main research objective is to identify, map, and synthesize the existing empirical literature on the design, use, and impact of boardgames to enhance life skills and well-being among inmates. To achieve this purpose, the following research questions will guide the study:*<br>*1. What empirical evidence is there on the design and impact of boardgames to improve life skills and well-being among inmates?*<br>*2. How are the different pedagogical, psychological, and technical factors addressed during the planning of boardgame activities for inmates?*<br>*3. What policy recommendations do derive from the collected evidence?*
**METHODS**		
Eligibility criteria	8	*The review follows the Population Concept Context (PCC) framework as criteria for eligibility for the review:*<br>*Population (P): Inmates;*<br>*Concepts (C): Use of boardgames to develop life skills and well-being among inmates;*<br>*Context: (C): Penitentiary institutions*<br>*All studies (research articles, evaluation reports, project reports, government reports, book chapters, ~~and~~ conference articles and qualitative studies) following PCC will be included according to the following criteria*<br>*a) are published from 2000 onwards.*<br>*b) are in Italian, English or Spanish languages.*<br>*c) address boardgames as life skills and well-being tools used among inmates.*
Information sources	9	*Electronic databases: PsycINFO, ERIC (Education Resources Information Center), SciELO, and Education Source, via Ebscohost simultaneously will be used as the search sources for this scoping review.*<br>*Separate searches will also be conducted via JSTOR, Emerald, Science Direct, DOAJ, IEEE Xplore, OECD Library, Springer Link, Taylor & Francis, ACM Digital Library. In addition, searches via Medline, Scopus, and Web of Science will be run, to cover additional journals not included within the above database.*<br>*Gray literature identification will be conducted in ProQuest, Google Scholar, and Semantic Scholar.*
Search strategy	10	*The bibliographic databases will be search using combination of the following search term, which are here formatted for the WOS database as a draft.*<br><br>*Search: Title*<br>*Years: from 2000 onwards*<br>*Languages: English OR Spanish OR Italian*<br>*1. TI=("boardgame" OR "boardgames" OR "board-game" OR "board-games" OR "board game" OR "board games" OR "COTS boardgame" OR "COTS boardgames" OR "COTS board-game" OR "COTS board-games" OR "COTS board game" OR "COTS board games" OR "Commercial Off-the-Shelf Game" OR "COTS Game" OR "COTS Games" OR "Commercial Off-the-Shelf Boardgame" OR "Gaming" OR "Gaming activity" OR "Game-based*

		learning" OR "Recreational Boardgame" OR "Role-Playing Game" OR "Role-Play Game" OR "strategy game" OR "Strategic game" OR "cooperative board games" OR "Serious games")<br>2. *TI=("Well-being" OR "Wellbeing" OR "Wellness" OR "Adaptive Behavior*" OR "Life Skills" OR "Self-Improvement")*<br>3. *TI=("Correctional setting*" OR "Correctional systems" OR "Correctional organizations" OR "Correctional institutions" OR "Community Correctional reentry centers" OR "Corrective systems" OR "Corrective settings" OR "Community corrections" OR "Prison systems" OR "Prison settings" OR "Penitentiary systems" OR "Penitentiary settings" OR "Custodial detentions")*<br>4. *TI=("Prisoner*" OR "Inmate*" OR "Jailbird*" OR "Detainee*" OR "Internee*" OR "Incarcerated" OR "Imprisoned" OR "Detained" OR "Convicted" OR "Criminals" OR "Condemned Inmates" OR "Offenders" OR "Felony offenders" OR "Locked up")*<br>5. *TI=("Correctional education" OR "Correctional Education Program*" OR "Community Corrections Program*" OR "Community Corrections Alternative Program" OR "Correctional practice" OR "Core Correctional practice" OR "Prison education" OR "Prisons Working Group" OR "Groupwork in Prisons" OR "Vocational Education and Training" OR "Soft Skills" OR "Non-formal learning" OR "Informal learning" OR "Formal learning" OR "Vocational education" OR "Vocational Training" OR "Vocational Education and Training" OR "Prison classroom*" OR "Skills Improvement" OR "European Skills Agenda*" OR "Lifelong Learning" OR "Qualification" OR "Self-Reliance" OR "Self-Esteem" OR "Self-Efficacy" OR "Life Skills Education" OR "Decision making" OR "Decision-making" OR "Problem solving" OR "Problem-solving" OR "Creative thinking" OR "Critical thinking" OR "Empathy")*
Study records:		
Data management	11a	*The management will be conducted using Microsoft Excel spreadsheet. An overview table will be created to display information about study characteristics from retrieved documents. This table will display information about location, author, year of publication, title, and sources.*
Selection process	11b	*Two reviewers will independently carry out the process of selection studies and, if any disagreement arises, it will be resolved via mutual discussion.*<br>*The search studies identify using the defined eligibility criteria (Item 8), will be included in the review under the following methodology:*<br>- *in the identification stage, the title will be screened for topic relevance.*<br>- *in the screening stage, the abstract will be read only if the title is in line with the objectives of the review.*<br>- *in the eligibility stage, the reviewers, referring to the PCC framework inclusion criteria, will independently read the full text of the studies selected.*
Data collection process	11c	*Data from the selected studies will be extracted according to the Joanna Briggs Institute (JBI) data extraction criteria. This process will be carried out in duplicate by two independent reviewers and a random sample of completed forms will be selected for inspection by the guarantor. Any discrepancies will be discussed until agreement will be reached.*<br>*Data extraction process will be carried out in tandem with validation from content experts.*
Data items	12	*The extracted data will be structured according to the Yusoff model, which includes nine conceptual variables to better*

		understand the different approaches used to design, develop, and implement boardgames, and to measure their impact on life skills improvements. The variables are: Capability; Instructional content; Intended learning outcomes; Game attributes; Learning activity; Reflection; Games genre; Game mechanics; Game achievement.
Outcomes and prioritization	13	*NA*.
Risk of bias in individual studies	14	*NA*
Data synthesis	15a	*We will employ a narrative strategy to summarize and synthetize data reported in the selected documents. The results will be presented using figures, concept maps, diagrams and tables.*
	15b	*NA*
	15c	*NA*
	15d	*NA*
Meta-bias(es)	16	*NA*
Confidence in cumulative evidence	17	*NA*

*** It is strongly recommended that this checklist be read in conjunction with the PRISMA-P Explanation and Elaboration (cite when available) for important clarification on the items. Amendments to a review protocol should be tracked and dated. The copyright for PRISMA-P (including checklist) is held by the PRISMA-P Group and is distributed under a Creative Commons Attribution Licence 4.0.**

From: Shamseer L, Moher D, Clarke M, Ghersi D, Liberati A, Petticrew M, Shekelle P, Stewart L, PRISMA-P Group. Preferred reporting items for systematic review and meta-analysis protocols (PRISMA-P) 2015: elaboration and explanation. BMJ. 2015 Jan 2;349(jan02 1):g7647.
