# Supplementary material for: Boardgames as an innovative approach to promote life skills and well-being among inmates: A scoping review protocol
Source: PLoS One. 2024 Feb 29;19(2):e0298728. doi: 10.1371/journal.pone.0298728 (PMC10903794; doi:10.1371/journal.pone.0298728)
Supplement: Attachment — Submitted filename: Response to Reviewers.docx [file pone.0298728.s003.docx]

Responses to each point raised by the Reviewers.

Reviewer#1: Introduction
Please give examples of boardgames and it will be nice to mention the ones intended for this scoping work. Board games as a word is too broad.

*R. We really thank the reviewer for showing interest in our research topic. We took inspiration from this observation to clarify the meaning of the term “boardgame” in the revised introduction of the re-submitted paper.*

*In few Italian detention facilities, located in the north and central part of Italy (Firenze, Genova, Imperia, Milano, Ravenna, Saluzzo, Sanremo) there are a variety of modern boardgames played in ongoing experimental activities with inmates. Here there is a list of some of the most frequently used: Cartagena, Stone Age, Carcassonne, Catan, Saboteur, Whitehall Mystery, Camel Up, Splendor, The Island, Ticket to Ride: Europe,7 Wonders Architects, Crossing, Project L, Azul, Flamme Rouge, For Sale, The Great Dalmuti, Avalon, The Resistance, Celestia, Cerberus, Deep Sea Adventure, etc.*

*On the BoardGameGeeks.com site is possible to find the technical details of each one of the boardgames listed above.*

*However, as reported in the submitted paper, our goal is to outline a scoping review protocol for mapping the literature on boardgames as tools for promoting life skills and well-being among inmates, in order to share with other researchers a standard approach to investigate and collect information around the world about which boardgames are in use, how they are used, what empirical evidence is there on the design and impact of boardgames to improve life skills and well-being among inmates, how are the diﬀerent pedagogical, psychological, and technical factors addressed during the planning of boardgame activities for inmates, what policy recommendations do derive from the collected evidence.*

*We respectfully consider it necessary to remain 'open' to any kind of boardgames we might find in use to be included in this work, especially because it is a Scoping Review. It seems necessary to stay open to all types of boardgames we will find, especially because the literature in this area is limited. This is evident from the fact that detention systems are enclosed and complex environments with unique limitations.*

Line 41-extensive (where?)

*R. We thank the reviewer for the encouraging clarification requested. Existing literature on boardgames in therapeutic contexts is extensive. We added few words and new references to the one already mentioned in the introduction of the re-submitted paper. Boardgames have been employed also in counseling process for decades, serving either as a supplementary tool or as a central component (Swank, J. M., & Weaver, J. L. (2021). In H. G. Kaduson & C. E. Schaefer (Eds.), Play therapy with children: Modalities for change (pp. 209–223). American Psychological Association. https://doi.org/10.1037/0000217-014; Bratton SC, Ray D, Rhine T, Jones L. The efficacy of play therapy with children: A meta-analytic review of treatment outcomes. Prof Psychol Res Pract 2005; 36:376–390.14; Nickerson ET, O’Laughlin KB. It’s fun—But will it work?:The use of games as a therapeutic medium for children and adolescents. J Clin Child Psychol 1980; 9:78–81.15; Wilde J. The effects of the Let’s Get Rational board game on rational thinking, depression, and self-acceptance in adolescents. J Ration Cogn Ther 1994; 12:189–196); (as reported in the revised introduction of the re-submitted paper).*

Line 42 -you mention other populations meaning they have been used in some populations-please mention them to offer context.
Generally, it is not very clear to me why the authors are interested in board games in prisons -please provide context of whether some formative work has been done and you want to prove hypothesis-there must always be reason for wanting to do something.

*R. Thank you again for the opportunity to better explain why we are interested in boardgames in prison. Research in this field could provide valuable insights into the potential benefits of boardgames for incarcerated populations, including their impact on social skills, mental health, and overall rehabilitation.*

*Examples of other populations include:*

*Juvenile Offenders: Research could explore how boardgames can be used as rehabilitative tools for juvenile offenders, fostering skill development, and reducing recidivism.*

*Adult Inmates in Correctional Facilities: Investigating the impact of boardgames on the social dynamics and mental well-being of adult inmates, promoting positive interactions and stress relief.*

*Inmates with Substance Abuse Issues: Exploring the potential of boardgames in addressing substance abuse issues among incarcerated individuals, complementing existing rehabilitation programs.*

*Women in Correctional Facilities: Studying how boardgames may contribute to the well-being and social integration of female inmates, considering gender-specific aspects of rehabilitation.*

*Inmates with Mental Health Conditions: Assessing the therapeutic benefits of boardgames for incarcerated individuals dealing with mental health challenges, potentially aiding in coping and rehabilitation.*

*Incarcerated Veterans: Researching the use of boardgames as a means of support and rehabilitation for incarcerated individuals who are military veterans, addressing their unique needs.*

*Long-Term Inmates: Examining the role of boardgames in maintaining mental stimulation and preventing social isolation among inmates serving long-term sentences.*

*Pre-Release Programs: Investigating the incorporation of boardgames into pre-release programs to prepare inmates for reintegration into society, focusing on social and cognitive skills.*

*Family Visitation Centers: Studying how boardgames can facilitate positive family interactions during visitation, contributing to the maintenance of family bonds despite incarceration.*

*Community Reentry Programs: Exploring the use of boardgames as part of community reentry programs, aiding in the transition from incarceration to society and promoting social integration.*

*The fundamental idea which drives our work is collecting concrete evidence regarding the potential benefits of boardgames in these specific populations. Through a scoping review that showcases existing literature, we intend to encourage governments, administrations, and any other entity/organization dealing with incarcerated individuals to consider of investing on a simple and cost-effective set of tools (boardgames). Through their ambivalence in effectiveness and benefits, these tools might contribute both to improving a higher level of 'humanity' in a rather critical context, to promoting well-being and to facilitating the development of life skills, aiming for an easier reintegration into social and work environments once the period of incarceration has concluded.*

References
The reference list is untidy, please tidy up.

*R. We thank the reviewer and we proceeded to tied it up with the integration of new updated references.*

Reviewer #2: You can use different keywords as the ones you used are already mentioned in the title

*R. We thank the reviewer, and we made the suggested changes.*

Please check for small typos (for example line 41-thei_r, line 72-diff_erent, line 108-"gray"->"grey").

*R. We thank the reviewer, and we check for the typos.*

Also, it would be more scientific if you used some references when you mention a potential problem that can occur during the study (for example line 175->...effectiveness (REF)., line 180->...among inmates (REF).

*R. We thank the reviewer, and we add the references respectively into the text of the re-submitted paper*.

In table 1, it would be useful to align the elements of the left column with those of the right.

*R. We thank the reviewer, and we took care of that.*

Finally, I would suggest making the legends of your tables and figures a little more descriptive to make them clear to the reader.

*R. We thank the reviewer, and we took care of that too as follows:*

*Tab 1* *The Population (or Participants)/Concept/Context (PCC) framework is recommended by the Joanna Briggs Institute (JBI) to identify the main concepts in primary review questions. The framework gives information about search strategy.*

*Fig 1:* *The PRISMA Flow Diagram for the Scoping Review process is a flowchart used to document the sources used in scoping reviews, and the selection and screening processes.*

*Fig 2:* *The Yusoﬀ, et al. Conceptual framework, which allows to clearly describe connections among the ideas about the effectiveness of boardgames as innovative interventions tools with positive outcomes and makes apparent the significance of the study.*
